# Moderate Grazing Promotes Fine Root Production in a Northern Saline–Alkaline Grassland

**DOI:** 10.3390/plants15091324

**Published:** 2026-04-26

**Authors:** Meng Cui, Congcong Zheng, Huajie Diao, Yingzhi Gao

**Affiliations:** 1 Key Laboratory of Grassland Resources and Ecology of Xinjiang Uygur Autonomous Region, College of Grassland Science, Xinjiang Agricultural University, Urumqi 830052, China; cuim141@nenu.edu.cn; 2 College of Grassland Science, Inner Mongolia Agricultural University, Hohhot 010011, China; 3 Institute of Computing Technology, Chinese Academy of Sciences, Beijing 100190, China; 4 College of Grassland Science, Shanxi Agricultural University, Taigu 030801, China

**Keywords:** grazing intensity, root production, root turnover, intermediate disturbance hypothesis, saline–alkaline grassland

## Abstract

Grasslands are key terrestrial ecosystems in which root dynamics regulate soil carbon and nutrient cycling. Although grazing constitutes the predominant land use practice in grassland ecosystems, its impacts on root dynamics remain inadequately elucidated, particularly across a gradient of grazing intensities. In this two-year field experiment, an improved root window method was applied to investigate the effects of four grazing intensities (no grazing, light grazing, moderate grazing, heavy grazing) on root production, root mortality, root standing crop, root turnover, and root lifespan in the saline–alkaline grassland in northern China. The results showed that root production and root mortality exhibited pronounced seasonal dynamics, with peaks in June and August for root production and in September for root mortality. These seasonal patterns were primarily driven by precipitation and were not significantly altered by grazing intensity. Moderate grazing significantly increased root production by 51.2% through changes in soil bulk density and selective livestock grazing, supporting the intermediate disturbance hypothesis. Root turnover was predominantly shaped by plant community composition and interannual precipitation, as opposed to grazing intensity. Overall, these findings indicate that moderate grazing promotes root growth, providing important insights into the sustainable utilization of saline–alkali grassland resources. In other words, appropriate measures must be taken to effectively manage grazing activities in the fragile saline–alkaline grasslands of northern China.

## 1. Introduction

As a critical part of terrestrial ecosystems, grasslands allocate a large share of their photosynthetic products to root systems [1,2,3]. Particularly in temperate grasslands, over 60% of photosynthetic assimilates are distributed to root systems, which constitute a key source of soil organic carbon within terrestrial ecosystems [2,4,5,6]. Accordingly, roots play a pivotal role in regulating soil carbon cycling processes [7,8]. However, due to the deep burial of roots beneath the soil surface and the difficulty in monitoring root production and root turnover, our understanding of the root growth progress has long been constrained.

Livestock grazing is the most widespread and long-standing management practice in grassland ecosystems, which directly or indirectly affects grassland ecosystems through trampling and foraging behaviors [2]. Until now, studies investigating how grazing affects grassland ecosystems have centered largely on aboveground net primary productivity (ANPP) [9,10], plant diversity [11,12,13], and community composition and structure [14,15], as well as nutrient cycling [16,17,18]. Numerous studies have explored grazing effects on root characteristics, such as root biomass [19,20,21], and belowground net primary productivity (BNPP) [22,23]. Nevertheless, few studies have examined the effects of grazing on root production and turnover.

Studies have shown that root responses to grazing are highly variable, including positive [24,25,26], negative [2,27,28], and neutral effects [29]. These inconsistent results suggest that root responses to grazing may depend on site conditions and soil physicochemical properties [3,30]. Overall, grazing reduces aboveground biomass and litter through foraging behavior, leading to increased soil temperature and decreased moisture, thereby inhibiting root production and enhancing root turnover [1,2,31]. Meanwhile, grazing often alters plant community structure through its foraging preferences [9,13,30]. On the other hand, grazing can also increase soil bulk density via trampling, and the increased mechanical resistance will further inhibit root production [23]. In addition, grazing intensity is commonly identified as a major factor leading to the disparate findings documented across studies [14,21,32]. The core tenet of the intermediate disturbance hypothesis proposes that ecosystem structure and functioning may reach a maximum at intermediate levels of disturbance, where competitive exclusion is reduced without causing severe stress to plants [33]. Moderate grazing is thought to stimulate compensatory plant growth via selective defoliation, thereby enhancing aboveground biomass [21,34,35,36], whereas overgrazing typically imposes strong inhibitory effects on plant growth [2,37]. However, owing to the scarcity of studies incorporating multiple grazing intensities within a single experiment, it remains unclear whether the intermediate disturbance hypothesis can also explain responses of root dynamics in grazed grassland ecosystems, particularly in saline–alkaline grasslands.

Grasslands represent the largest terrestrial ecosystem in China, accounting for approximately 40% of the country’s total area (3.93 million km^2^) [38]. The area of grassland salinization caused by long-term overgrazing is gradually expanding [39,40,41,42], and it exerts negative impacts on plant growth by deteriorating the soil environment [43,44]. Consequently, defining the ideal grazing intensity that harmonizes economic gains with the sustainable development of grassland ecosystems is now a key priority for current grassland management practices, particularly in northern saline–alkaline grasslands where ecosystem resilience is inherently constrained by soil salinity and alkalinity.

To address this critical research gap, we conducted a field experiment in a saline–alkali grassland in northern China to evaluate the effects of different grazing intensities on root production and turnover. Briefly, we aimed to identify which grazing intensity can enhance root production and turnover, which is of great significance for the sustainable use of grassland resources. Specifically, we aim to address the following research questions: (1) How do varying grazing intensities affect root seasonal dynamics, production, and turnover, and what are the potential mechanisms? (2) Does the intermediate disturbance hypothesis apply to root production and turnover in saline–alkaline grasslands?

## 2. Materials and Methods

### 2.1. Study Area

The experiment was conducted at the Youyu Loess Plateau Grassland Ecosystem National Research Station (39°59′ N, 112°19′ E, altitude: 1348 m), located in Youyu County, Shanxi Province, northern China. Over the past three decades, the mean annual air temperature was 4.6 °C, and the mean annual precipitation totaled 425 mm, with rainfall events predominantly occurring in July and August. During the two consecutive experimental years, there was no significant difference in mean annual temperature, but precipitation differed significantly: precipitation in 2018 was 490 mm, whereas in 2019, it was only 310 mm (Figure S1). The vegetation at the study site is mainly composed of *Leymus secalinus* (Georgi) Tzvel., *Puccinellia tenuiflora* (Griseb.) Scribn. & Merr., and mixed forb assemblages. In particular, as a perennial rhizomatous grass, *Leymus secalinus* has become the absolute dominant species in this region due to its drought and saline–alkali tolerance, contributing nearly 60% of the total aboveground productivity. *Puccinellia tenuiflora* is a perennial, tufted grass species with extremely high saline–alkali tolerance but relatively weak drought resistance. As a typical dominant species in saline–alkali grasslands of China, it often grows in association with species such as *Leymus secalinus*. In accordance with the Chinese Soil Taxonomy, the study site’s soil is categorized as chestnut soil, and its pH and electrical conductivity (EC) are determined to be 9.0 and 3.4 ms cm^−1^ [45]. Soil total N, soil total P, and soil organic C of topsoil (0–10 cm) are 0.8 g kg^−1^, 0.39 g kg^−1^, and 5.43 g kg^−1^, respectively [46].

### 2.2. Experimental Treatment and Design

A randomized block design was employed in the present study, with four grazing intensity levels set as follows: no grazing (G0), light grazing (G1, 2.35 sheep units·hm^−2^·growing season^−1^), moderate grazing (G2, 4.80 sheep units·hm^−2^·growing season^−1^), and heavy grazing (G3, 7.35 sheep units·hm^−2^·growing season^−1^). Four replications were assigned to each treatment, giving a total of 16 experimental plots that were uniformly rectangular and measured 25 m × 40 m (1000 m^2^).

### 2.3. Sample Collection

#### 2.3.1. Measurements of Root Dynamics

We assessed root dynamics via the improved root window method [1,2,47], and in September 2017, one vertical glass root window (28 cm × 20 cm × 0.3 cm; height × length × thickness) was installed in each plot. Two 10 × 10 cm squares were marked on each glass window by carving 2 mm wide grooves, positioned 8 cm above the bottom and 5 cm from both lateral edges. Dark iron sheets (20 cm × 2 cm, length × width) were applied to seal soil–glass gaps and eliminate light disturbance to root growth. A vertical soil profile was dug in each plot to fit the glass root window, which was firmly pressed against the excavation face and stabilized with iron stakes on either side of the window. Following installation, the excavated soil was backfilled and compacted to restore the original soil bulk density. The glass windows remained undisturbed throughout the experimental period.

Root growth was monitored with an HP Scanjet G2410 scanner (Hewlett-Packard, Palo Alto, CA, USA) placed roughly 1 cm away from the root windows. Initiated on 2 May 2018, roughly eight months after the glass root windows were installed, scanning was conducted at 15-day intervals through to 19 October 2019. On each sampling occasion, the backfilled soil was cautiously removed, and the glass surface was lightly wiped clean using a soft cloth. After image capture, the soil was carefully repositioned and compacted following the same protocol as mentioned above.

Root appearance and disappearance were analyzed with MapInfo Professional 7.0 (Pitney Bowes MapInfo Corporation, New York, NY, USA). Each individual root observed in the baseline images was assigned a distinct identification code. Images collected at successive sampling dates were compared against the initial images to distinguish original roots from newly produced individuals, with newly emerged roots also assigned corresponding identification codes. Roots that were no longer detectable in later scans were categorized as dead or decomposed. In detail, root production per interval was calculated as the combined length of new lateral branches emerging from existing roots and newly initiated roots in total. Root mortality was assessed by quantifying the total length of roots that senesced or vanished during each measurement period. Root standing crop was derived from the disparity between cumulative root production and cumulative root mortality across the growing season, while root longevity was defined as the days elapsed between a root’s first detection and its eventual disappearance.

#### 2.3.2. Soil Moisture

A portable TDR100 time-domain reflectometer (Campbell Scientific, Bremen, Germany) with a 7.5 cm probe was used to measure volumetric soil water content (% V) in the top 0–10 cm soil layer. Measurements were taken at 15-day intervals, synchronized with root scanning dates. Within each plot, five replicate measurements were collected adjacent to the root window, and their mean value was used for subsequent statistical analysis.

### 2.4. Data Analysis

We tested the normality and homoscedasticity of the dataset with the Shapiro–Wilk test prior to further analysis. Two-way ANOVA was employed to evaluate how grazing intensity, soil layer, and their interaction influence root production, mortality, standing crop, and turnover; where significant interactive effects were detected, one-way ANOVA was applied to quantify the independent contributions of each variable. Kaplan–Meier survival analysis was used to estimate the mean root lifespan. To quantify the relative importance of biotic and abiotic factors on root production and root turnover, a random forest (RF) model was performed using the randomForest package in R [48]. A total of 4 explanatory variables were included in the model, namely, soil bulk density (SD), soil temperature (ST), soil water content (SWC), and the relative biomass percentage of the dominant species, *Leymus secalinus*, which represented selective grazing intensity. The model was run with 5000 decision trees to ensure stability, and the percentage increase in mean squared error (%IncMSE) was used to evaluate the relative importance of each predictor. Higher %IncMSE values indicated greater contributions of the corresponding variable to the observed variation in root production and root turnover.

Statistical analyses were executed in R v4.5.1 (R Core Team, 2025) via the R Studio interface (v1.3.1056). The tidyverse, dplyr, and ggplot2 libraries were applied for data wrangling and visualizing root dynamic indices, and the OIsurv and ggplot2 packages were used to quantify and visualize root lifespan patterns [49,50,51].

## 3. Results

### 3.1. The Seasonal Dynamics on Root Production, Mortality, and Standing Crop

**Figure 1:**
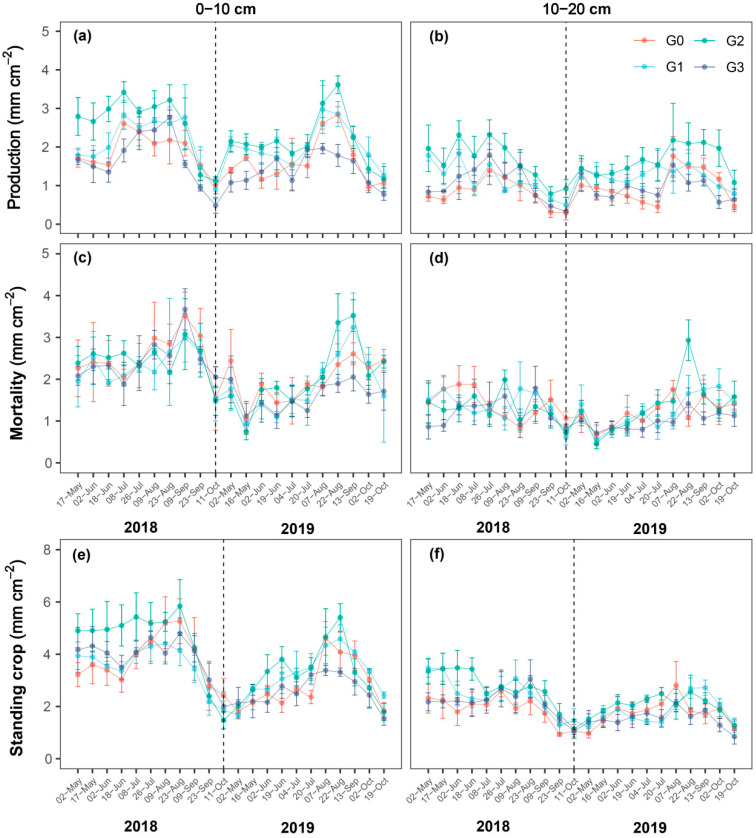
Seasonal dynamics of root production, root mortality, and mean root standing crop under different grazing intensities and soil layers. Data are mean ± SE (*n* = 4) in the 0–10 cm and 10–20 cm soil layer during the growing seasons from 2 May 2018 to 19 October 2019. G0, G1, G2, and G3 represent no grazing, light grazing, moderate grazing, and heavy grazing. (**a**) root production in 0–10 cm; (**b**) root production in 10–20 cm; (**c**) root mortality in 0–10 cm; (**d**) root mortality in 10–20 cm; (**e**) root standing crop in 0–10 cm; (**f**) root standing crop in 10–20 cm.

Root production and mortality of the plant community in northern China’s saline–alkaline grasslands exhibited distinct seasonal dynamics, which were primarily driven by precipitation. Overall, root production reached its peak in June and August across all grazing treatments, and different grazing intensities did not significantly modify the seasonal pattern of root production (Figure 1a,b). In contrast, root mortality attained its seasonal maximum in September (Figure 1c,d). These seasonal fluctuations were more prominent in the 0–10 cm soil layer but less so in the 10–20 cm layer. Root standing crop, an indicator of the overall balance between root production and mortality, gradually declined after peaking in August (Figure 1e,f).

### 3.2. Root Production, Root Mortality, and Root Standing Crop

**Figure 2:**
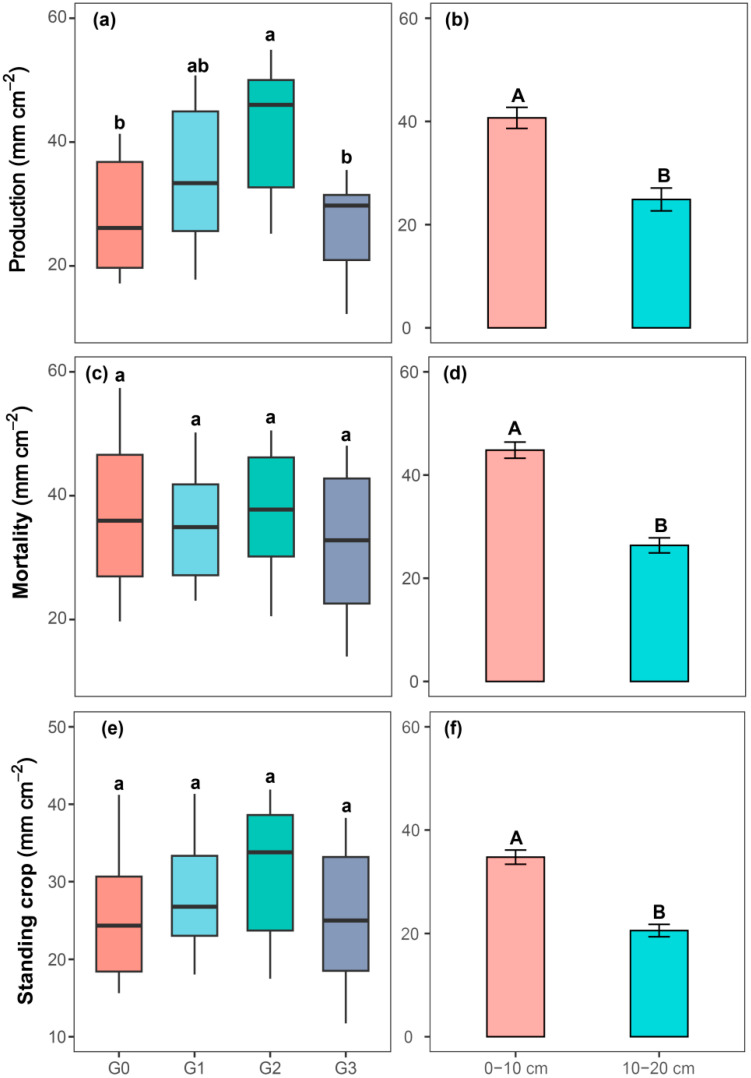
Cumulative root production (**a**,**b**), cumulative root mortality (**c**,**d**), and mean root standing crop (**e**,**f**) under different grazing intensities and soil layers during the growing seasons from 2 May 2018 to 19 October 2019. Data are mean ± SE with *n* = 4. Different lowercase letters above the boxplots indicate significant differences among different grazing intensities. Different uppercase letters above the bar charts indicate significant differences among different soil layers (*p* < 0.05). G0, G1, G2, and G3 represent no grazing, light grazing, moderate grazing, and heavy grazing.

Across this two-year field trial, cumulative root production and mortality were recorded at 12.2–54.9 mm cm^−2^ and 14.2–57.3 mm cm^−2^, respectively. Root production was significantly affected by grazing intensity and soil layer, with no statistically significant interactive effect between these two factors. Root mortality, however, was only significantly impacted by soil layer (*p* < 0.05, Table 1). For the 0–20 cm soil profile during the experimental period (2 May 2018–19 October 2019), moderate grazing (G2) yielded the highest root production, which was 51.2% and 58.1% greater than the no-grazing (G0) and heavy grazing (G3) treatments, respectively (Figure 2a). A significant soil layer effect was observed for root production, with the 0–10 cm layer having a mean production 63.6% higher than the 10–20 cm layer (Figure 2b). Root mortality showed no significant variation across the gradient of grazing regimes (Figure 2c). Similar to root production, the soil layer had a significant effect on root mortality, with the 0–10 cm layer exhibiting a 70% higher mortality rate than the 10–20 cm layer (Figure 2d).

### 3.3. Annual Root Turnover and Mean Root Lifespan

**Figure 3:**
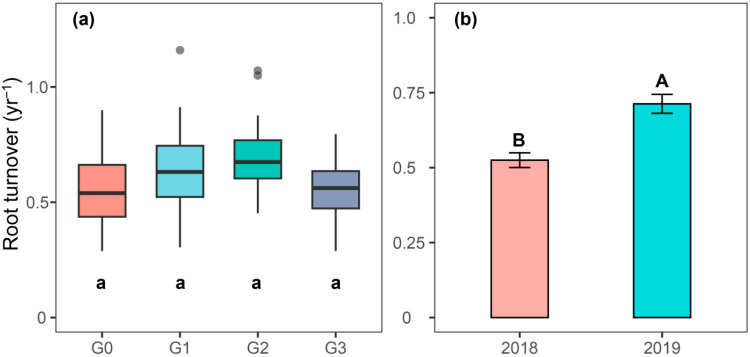
Annual root turnover (times yr^−1^, mean ± SE) of 0–20 cm at different grazing intensities (**a**) and different years (**b**). Different lowercase letters above the boxplots indicate significant differences among different grazing intensities. Different uppercase letters above the bar charts indicate significant differences among different years (*p* < 0.05). G0, G1, G2, and G3 represent no grazing, light grazing, moderate grazing, and heavy grazing.

Root turnover rates during the two-year study spanned 0.29 to 1.2 yr^−1^, with grazing intensity and year both exerting significant impacts on this parameter (*p* < 0.05, Table S1). No significant statistical differences were found across grazing treatments; however, root turnover under moderate grazing (G2) achieved the highest value of 0.71 yr^−1^ (Figure 3a). Root turnover differed significantly between the two years: the 2018 rate was notably lower, averaging 35.8% less than the 2019 rate (Figure 3b).

**Figure 4:**
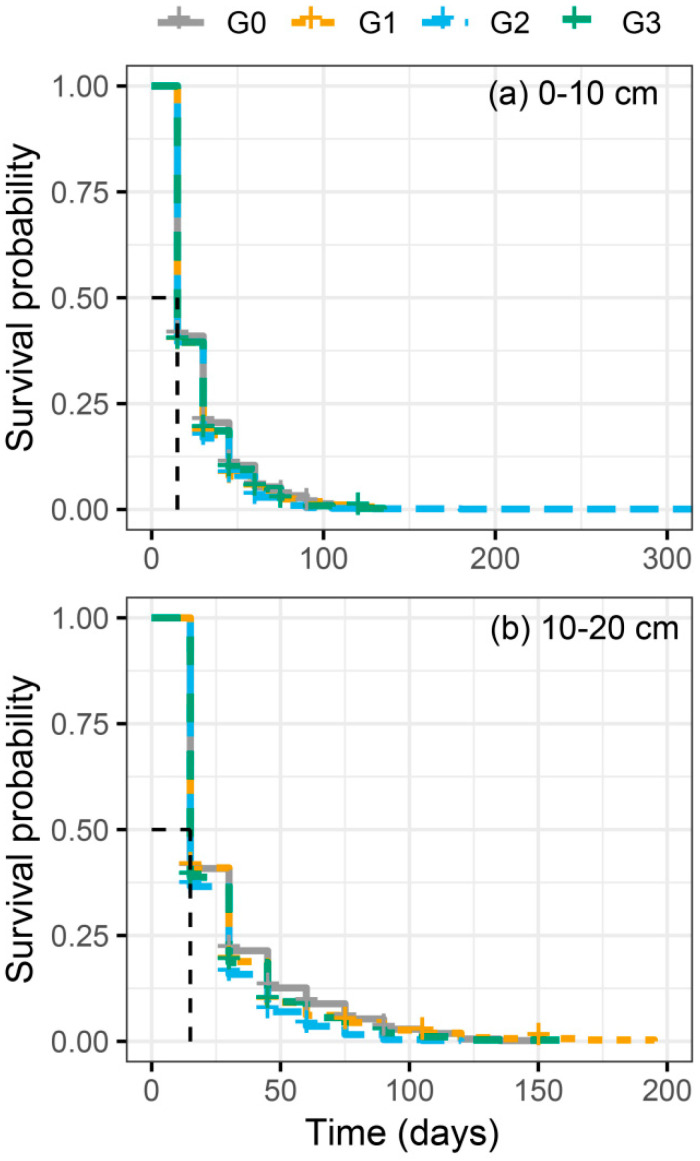
Survival curves for cohorts of roots arising in the 0–10 cm (**a**) and 10–20 cm (**b**) soil depths within the different grazing intensities. The mean and median lifespan were calculated via the survival curves using Kaplan–Meier analysis. The log-rank test was performed to compare the effects of different treatments on the root survival rate. G0, G1, G2, and G3 represent no grazing, light grazing, moderate grazing, and heavy grazing. The dashed line represents the median survival time of roots.

Grazing intensity had no significant influence on root lifespan in the 0–10 cm soil layer (*p* > 0.05). Under no grazing (G0), the mean root lifespan was the longest, at 26.25 days. More than 60% of roots survived for less than 20 days, and nearly all roots had died by day 90. Regarding root survival probability, G0 achieved the highest rate, while G2 had the lowest. It is worth noting that the maximum root lifespan was recorded under G2, reaching 310 days (Figure 4a). In contrast, in the 10–20 cm soil layer, grazing treatments significantly affected root lifespan (*p* < 0.05): G0 had the highest root survival rate and the longest mean lifespan, while G2 remained the treatment with the lowest root survival rate (Figure 4b).

### 3.4. Driving Factors of Root Production Affected by Grazing

**Figure 5:**
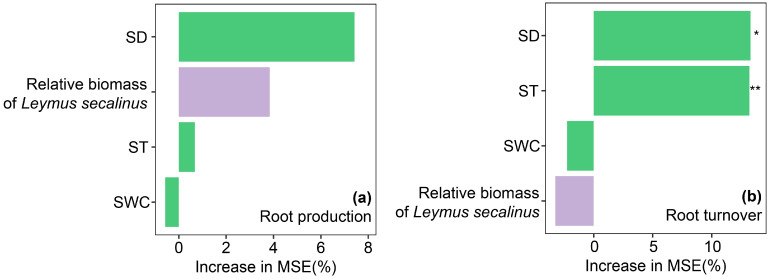
The drivers of root production (**a**) and root turnover (**b**) in different grazing treatments. The importance of the factors represented by the mean square error (% IncMSE) of different grazing treatments for biological factors (green) and soil factors (purple). SD stands for soil bulk density, ST for soil temperature, and SWC for soil water content. Statistical significance is depicted as: *, 0.01 < *p* < 0.05; **, 0.001 < *p* < 0.01.

Soil factors exhibited nonlinear relationships with root production and turnover. Specifically, root production showed a significant nonlinear response to soil bulk density, increasing initially and then declining with increasing bulk density (R^2^ = 0.22, *p* < 0.05; Figure S3a), but it showed no significant correlation with soil temperature or soil water content (*p* > 0.05; Figure S3b,c). For root turnover, a significant nonlinear relationship with soil bulk density was also observed (R^2^ = 0.33, *p* < 0.01; Figure S3d). Root turnover declined approximately linearly with increasing soil temperature (R^2^ = 0.20, *p* < 0.05; Figure S3e). Similar to root production, root turnover was not significantly correlated with soil water content (*p* > 0.05; Figure S3f). In addition, we conducted a random forest analysis based on the data in this experiment. Among all factors, we found that the soil bulk density was the most important factor affecting root production under different grazing treatments, and the relative biomass percentage of *Leymus secalinus* was also the second most important factor (Figure 5a). For root turnover, soil bulk density and soil temperature were the top two influencing factors (Figure 5b).

## 4. Discussion

### 4.1. The Root Dynamics Pattern

As the dominant contributor to soil organic matter, roots serve a crucial function in connecting aboveground vegetation with the soil environment [7,52]. Grazing represents the dominant grassland management regime and can profoundly affect soil nutrient cycling in grassland ecosystems [53,54]. However, research investigating how grazing influences root dynamics in grassland ecosystems, especially along an intensity gradient, remains relatively scarce [9,11]. In the present research, the improved root window method was utilized for in situ monitoring of root dynamics, and we assessed how varying grazing intensities influence root production, mortality, standing crop, and turnover in the northern China saline–alkali grassland. Our results demonstrated that root production and mortality exhibited distinct seasonal patterns. These seasonal patterns were not altered by different grazing intensities. Specifically, root production peaked in June and August (Figure 1a,b). However, this spring peak was absent in 2019, a year characterized by relatively low spring precipitation (Figure S1). The spring peak in fine root production is likely associated with rising soil temperature and enhanced photosynthetic activity accompanying leaf expansion [1,51]. Compared with 2019, this spring peak was more strongly coupled with spring precipitation (Figure 1a,b and Figure S1). By contrast, the autumn peak in fine root production was mainly attributed to the dominant perennial species (e.g., *Leymus secalinus*), which allocates additional resources to belowground nutrient storage after seed reproduction to support growth in the following year. In contrast to root production, root mortality exhibited only one distinct peak in autumn (Figure 1c,d). Previous studies have reported that root production and mortality often occur synchronously and are strongly positively correlated, leading to similar peaks in root mortality and standing crop [2,55,56]. However, inconsistent with these findings, we observed no spring peak in root mortality. This discrepancy may reflect a plant strategy of retaining older roots and reducing root mortality early in the growing season, thereby allowing greater allocation of photosynthates to aboveground tissue [2,47]. Furthermore, our findings revealed that the seasonal patterns of root dynamics were not significantly influenced by grazing intensity, suggesting that seasonal changes in root traits in this region are mainly regulated by climatic factors (e.g., precipitation) and plant community composition, rather than by grazing.

Our study revealed that root production in the northern Chinese saline–alkaline grasslands varied between 12.2 and 54.9 mm·cm^−2^. This finding aligns with the research results reported by Bai et al. (2015) and Cui et al. (2024) [1,2]. On the other hand, Wu et al. (2020) documented a higher level of root production in the alpine meadows of the Qinghai–Tibet Plateau [57]. The observed inconsistencies between these studies may be attributed to variations in vegetation composition, climatic regimes, soil characteristics, and root monitoring techniques across different study locations [1,47,58]. The Qinghai–Tibet Plateau, characterized by its high altitude, is dominated by plant species such as *Kobresia humilis* and *Festuca ovina*; these low-growing species generally have higher root production than the dominant species in our study region, including *Leymus secalinus* [59]. According to the Chinese Soil Taxonomy, the soil type of alpine meadows is classified as *Mat Cryic Cambisol*. In addition, both mean annual temperature and precipitation are lower than in our research region. Under such harsh climatic conditions, plants typically develop denser root systems to secure adequate water and nutrient uptake for regular growth and survival [60]. Notably, the minirhizotron adopted by Wu et al. [57] is distinct from the one we used in the present research. This difference in methodology may represent an important reason for the inconsistent results. Compared with traditional destructive root sampling methods, the in situ observation technique used in our study can effectively reduce the loss of fine roots during measurement [1]. Importantly, root growth can continue even during the non-growing season, especially in regions dominated by perennial plant species [3,4]. Therefore, both the minirhizotron method and the improved root windows method are widely accepted because of their ability to support continuous root monitoring. However, the minirhizotron method usually covers a relatively small monitoring area, which often leads to the overestimation of root production [2,47].

### 4.2. Responses of Root Production and Turnover to Different Grazing Intensities

Across different grazing intensity gradients, our results indicated that moderate grazing notably increased root production-this finding provides additional support for the intermediate disturbance hypothesis (Figure 2). The underlying mechanisms explaining this response are as follows. First, under moderate grazing, livestock trampling caused a slight increase in soil bulk density, which generated moderate mechanical resistance for root growth (Figure 5 and Figure S2). To support vigorous aboveground growth, plants correspondingly increased root production [61,62]. However, under heavy grazing, soil bulk density increased further, and excessive mechanical resistance restricted root elongation and growth [63]. Second, moderate grazing stimulated plants to enhance root growth to facilitate faster recovery of aboveground tissues. Specifically, under moderate grazing intensity, livestock selectively foraged and reduced the relative biomass of annual species, which enhanced the competitive advantage of the dominant species, *Leymus secalinus,* in the study area, thereby increasing root production (Figure 5 and Figure S4). In contrast, under heavy grazing, severe loss of aboveground photosynthetic tissues limited the supply of photosynthates to belowground parts. Under this stress, plants allocated more photosynthetic products to aboveground structures and maintained or reduced carbon allocation to roots, which resulted in lower root production. Our results showed that precipitation did not have a significant impact on root production during the two consecutive experimental years (Figure 3a). This result may be attributed to the inhibitory effect of excessive precipitation during the 2018 growing season on root growth (Figure S1). Nevertheless, root production in the 0–10 cm soil layer was consistently higher than in the 10–20 cm layer across all grazing treatments (Figure 3b). This pattern was mainly driven by vegetation composition. In addition to the dominant species, *Leymus secalinus*, numerous annual species, such as *Potentilla anserina*, *Saussurea japonica*, and *Taraxacum mongolicum*, occurred in the study area (Figure S4), and their roots were mainly concentrated in the topsoil. In addition, more favorable environmental conditions in the surface soil may also be a potential factor contributing to the high proportion of roots in the 0–10 cm soil layer [64].

It is broadly acknowledged that root turnover rate serves as a critical metric for assessing carbon partitioning and nutrient cycling processes in grassland ecosystems and also acts as an important reference for understanding the allocation strategies of photosynthates. Generally, slower root turnover indicates lower nutrient use efficiency of plant roots in soil [1,65]. Our results showed that grazing intensity had no significant effect on root turnover, regardless of year and soil layer (Figure 3). This pattern was mainly attributed to the dominance of *Leymus secalinus* in this area. Many previous studies have demonstrated that root turnover exhibits relatively small variation within the same plant species, which masks the effects of different grazing intensities [47,66]. In other words, grassland ecosystems dominated by perennial plant species exhibit greater resistance to grazing disturbance compared to those dominated by annual or biennial species [67,68,69]. Furthermore, our findings revealed that within the 0–10 cm soil layer, G2 resulted in the longest root lifespan while yielding the lowest root survival rate (Figure 4a). A possible explanation for this phenomenon is that grazing animals selectively forage on annual and biennial plants. This causes widespread root mortality in the surface 0–10 cm soil layer, where most fine roots are distributed, and thus lowers root survival rate [15,34,70]. At the same time, under moderate grazing intensity, the dominant perennial grass *Leymus secalinus* adopts a strategy of prolonging root lifespan to reduce the allocation of photosynthetic products to roots so as to ensure the recovery of aboveground parts.

## 5. Conclusions

Grazing is the dominant land use type in global grasslands. Clarifying root responses to grazing is crucial for the sustainable management of fragile saline–alkali grasslands. Our study shows that root seasonal dynamics were unaffected by grazing intensity but strongly controlled by precipitation. Moderate grazing increased root production by 51.2% and extended root lifespan via changes in soil bulk density and selective grazing, whereas root turnover was mainly driven by plant community composition and annual precipitation. Overall, moderate grazing promoted root production while maintaining natural root dynamics, supporting the intermediate disturbance hypothesis. For grassland management, moderate grazing represents an effective strategy to balance root productivity and ecosystem function in northern saline–alkali grasslands. Despite the limitations of this experiment, namely, the relatively high variability in the results caused by only four grazing intensity treatments and a total of 16 experimental plots, our study underscores the significant role of moderate grazing in promoting root productivity within grassland ecosystems. These findings provide critical empirical evidence for sustainable grazing management and the conservation of such ecosystems under global change. More importantly, future research is warranted to further explore the diverse response patterns of root systems and their underlying driving mechanisms, particularly in fragile habitat types.

## Figures and Tables

**Table 1:** Results of two-way ANOVA for effects of grazing intensities (G), soil layers (S), and their interactions (G*S) on root production, root mortality, and root standing crop. Statistical significance results are presented directly in the table, where ns indicates no significant difference.

		Root Production		Root Mortality		Root Standing Crop	
	df	*F*	Sig.	*F*	Sig.	*F*	Sig.
G	3	10.67	<0.001	1.02	0.4 ns	2.33	0.09 ns
S	1	52.69	<0.001	67.91	<0.001	62.95	<0.001
G*S	3	0.45	0.71 ns	0.15	0.92 ns	0.1	0.96 ns

## Data Availability

Data are contained within the article and Supplementary Materials.

## Supplementary Materials

The following supporting information can be downloaded at: https://www.mdpi.com/article/10.3390/plants15091324/s1, Figure S1. The annual climate variability of the region from 2018 to 2019. Values on each panel are total annual precipitation and mean temperature; Figure S2. Soil bulk density (a,b), soil temperature (c,d), and soil water content (e,f) under different grazing intensities from 2018 to 2019. Different lowercase letters indicate significant differences among different grazing intensities. G0, G1, G2, G3 represent no grazing, light grazing, moderate grazing, heavy grazing; Figure S3. Regressions of root production and root turnover on soil bulk density (a,d), soil temperature (b,e) and soil water content (c,f). Data for root production were the cumulative values in the 0–20 cm soil layer from a two-year consecutive field experiment. The data of soil bulk density, soil temperature and soil water content were those mean values in the experimental plots in the top soil depths during the growing seasons across two years; Figure S4. Species relative biomass under different grazing intensities from 2018 to 2019. G0, G1, G2, G3 represent no grazing, light grazing, moderate grazing, heavy grazing; Table S1. Results of three-ANOVA for effects of grazing intensities (G), soil layers (S), year (Y) and their interactions on root turnover.
