# Supplementary material for: Moderate Grazing Promotes Fine Root Production in a Northern Saline–Alkaline Grassland
Source: Plants (Basel). 2026 Apr 26;15(9):1324. doi: 10.3390/plants15091324 (PMC13165009; doi:10.3390/plants15091324)
Supplement: Supplementary file 1 [file plants-15-01324-s001.zip › plants-4240811-supplementary.pdf]

Supplemental Figure S1. The annual climate variability of the region from 2018 to 2019. Values on each panel are total annual precipitation and mean temperature.

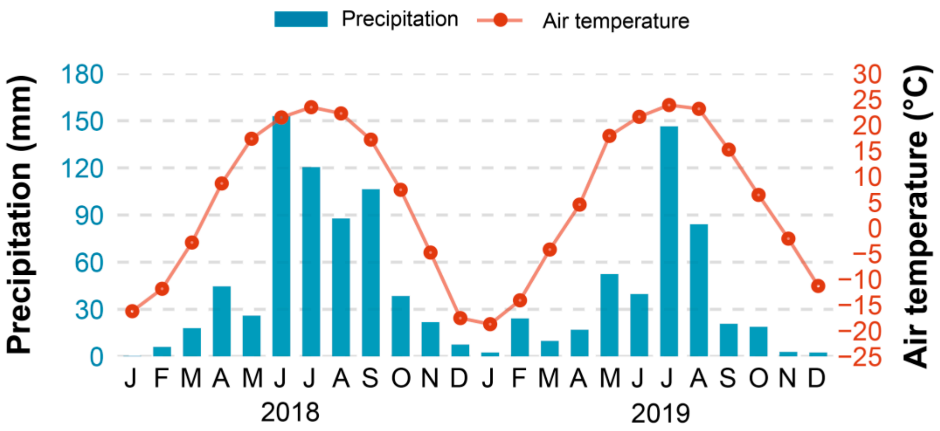

Supplemental Figure S2. Soil bulk density (a, b), soil temperature (c, d), and soil water content (e, f) under different grazing intensities from 2018 to 2019. Different lowercase letters indicate significant differences among different grazing intensities. G0, G1, G2, G3 represent no grazing, light grazing, moderate grazing, heavy grazing.

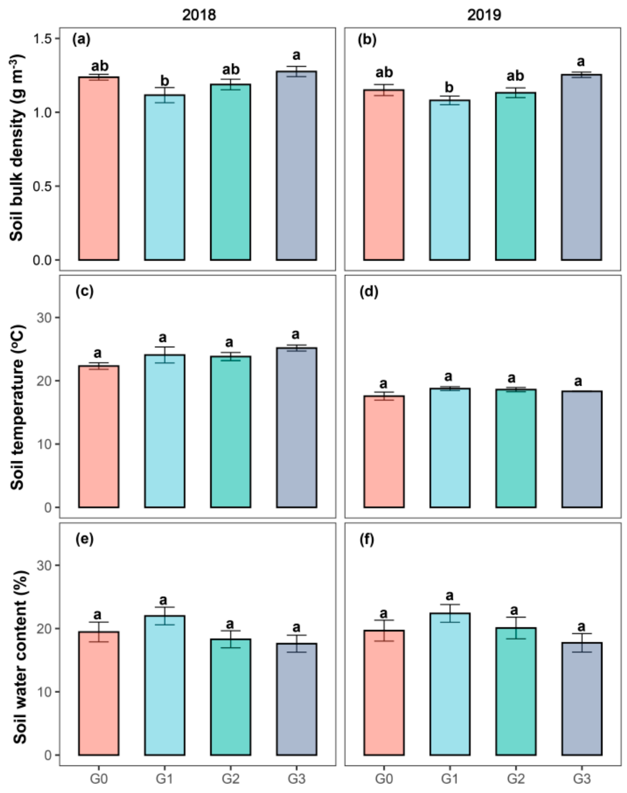

Supplemental Figure S3. Regressions of root production and root turnover on soil bulk density (a, d), soil temperature (b, e) and soil water content (c, f). Data for root production were the cumulative values in the 0-20 cm soil layer from a two-year consecutive field experiment. The data of soil bulk density, soil temperature and soil water content were those mean values in the experimental plots in the top soil depths during the growing seasons across two years.

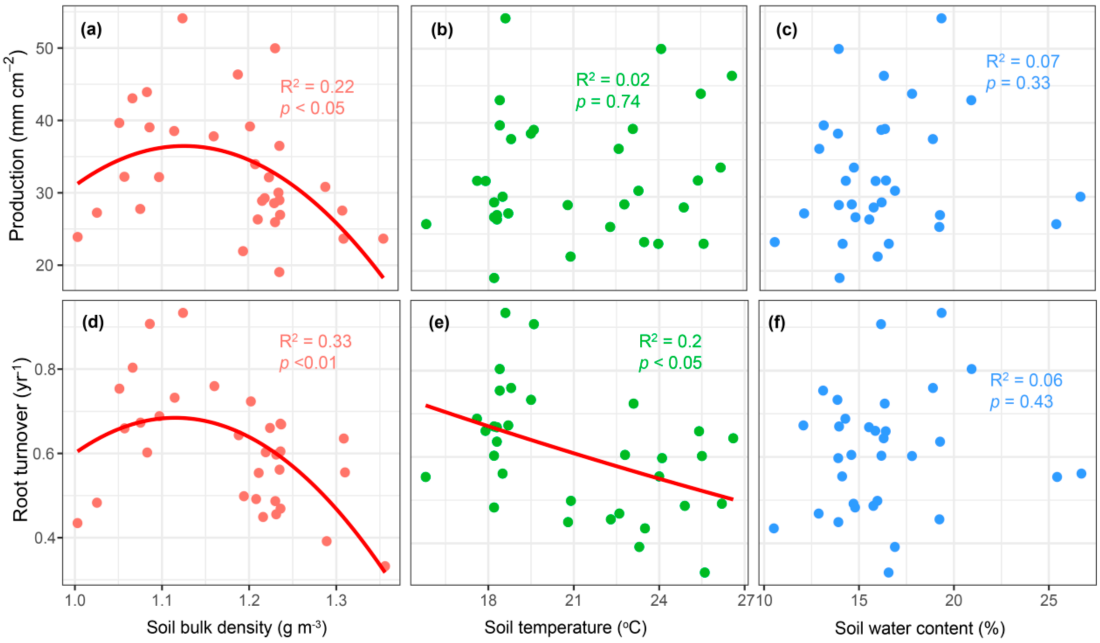

Supplemental Figure S4. Species relative biomass under different grazing intensities from 2018 to 2019. G0, G1, G2, G3 represent no grazing, light grazing, moderate grazing, heavy grazing.

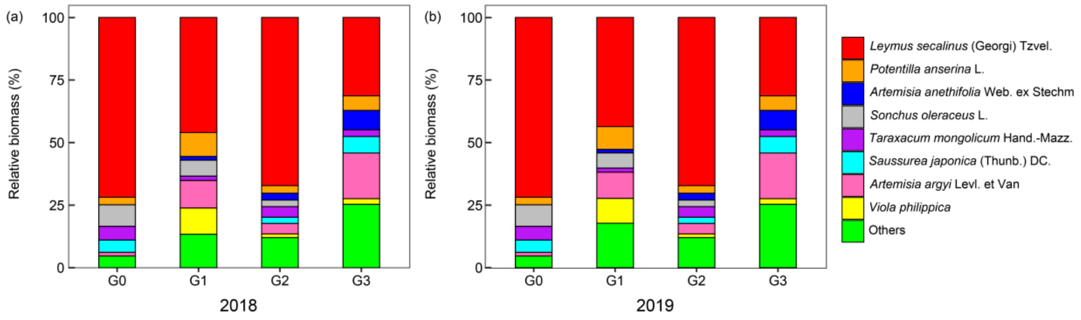

Table S1: Results of three-ANOVA for effects of grazing intensities (G), soil layers (S), year (Y) and their interactions on root turnover.

		Root turnover	
	df	*F*	Sig.
G	3	3.11	<0.05
S	1	0.51	0.48ns
Y	1	22.21	<0.001
G*S	3	1.85	0.18ns
Y*S	1	0.56	0.65ns
G*Y	3	0.08	0.97ns
G*S*Y	3	0.25	0.86ns
